# Evaluating the methodological suitability of partial dependence plots and Shapley additive explanations for population-level interpretation of machine learning models in total joint arthroplasty

**DOI:** 10.1186/s42836-025-00360-9

**Published:** 2026-01-29

**Authors:** Kole Joachim, Othneil Sparks, Amanda Perrotta, Adrian Lin, Brandon Gettleman, Christopher Hamad, Sumin Jeong, Ezekiel Dingle, Alexandra Stavrakis, Alexander B. Christ

**Affiliations:** 1 David Geffen School of Medicine, University of California, Los Angeles, CA 90095 USA; 2 Department of Orthopaedic Surgery, University of California, Los Angeles, CA 90095 USA; 3 Department of Orthopaedic Surgery, Greater Los Angeles VA Medical Center, Los Angeles, CA 90073 USA

**Keywords:** Total joint arthroplasty, Machine learning, Interpretability, Partial dependence plots, SHAP, Risk prediction, Postoperative complications

## Abstract

**Background:**

Total joint arthroplasty (TJA) complications necessitate the development of accurate risk prediction models; however, interpretability in machine learning remains a challenge. While Shapley Additive Explanations (SHAP) offers insights at the individual level, partial dependence plots (PDPs) may provide a better understanding at the population level for developing clinical guidelines. This study compared PDPs and SHAP in explaining machine learning-based 30-day complication risk prediction following TJA.

**Methods:**

We conducted a retrospective cohort study using the American College of Surgeons National Surgical Quality Improvement Program (NSQIP) database (2019–2023), including 517,826 primary TJA cases. Binary classification models (Random Forest, Gradient Boosting) predicted composite 30-day complications based on 20 clinical predictors. A comprehensive interpretability analysis employed directional concordance validation between PDP and SHAP, permutation importance thresholding (5% relative influence), followed by one- and two-dimensional partial dependence analyses with explicit interaction modeling.

**Results:**

The cohort comprised 517,826 primary TJA procedures with a complication rate of 6.67%. The baseline Random Forest model achieved test AUC = 0.678. Directional concordance analysis demonstrated 97.8% weighted agreement between PDP trends and SHAP attributions, validating methodological comparison. Threshold analysis identified seven significant features, with interaction effects accounting for 49.9% of total model influence (71.9% among top features). PDPs showed actionable dose–response relationships, including critical thresholds for preoperative hematocrit (< 38%), operative time (> 120 min), and complementary interactions, such as age × ASA classification (19.1% importance), operative time × ASA classification (10.1%), and hematocrit × diabetes (6.4%). Comparative patient analysis demonstrated that while SHAP quantified individual contributions, only PDPs provided population thresholds directly translatable to institutional protocols.

**Conclusion:**

PDPs appear more methodologically appropriate than SHAP for population-level clinical guideline development, offering actionable dose–response relationships and population risk thresholds that SHAP’s individualized attribution framework cannot provide. The dominance of interaction effects among the most influential predictors validates that PDPs accurately capture complementary relationships while presenting them in a format directly applicable to evidence-based perioperative protocols and institutional quality improvement initiatives.

Video Abstract

**Supplementary Information:**

The online version contains supplementary material available at 10.1186/s42836-025-00360-9.

## Introduction

Total joint arthroplasty (TJA), including total hip (THA) and knee arthroplasty (TKA), is a highly effective treatment for end-stage joint disease [1]. However, TJA carries the risk of significant postoperative complications, such as surgical site infections, venous thromboembolism, and unplanned readmissions or reoperations [2–5]. Accurate risk prediction for these complications is crucial for enhancing patient outcomes and optimizing resource management. Therefore, the complexity and diversity of TJA populations require interpretable predictive models to build clinical trust and utility [6–9]. Machine learning (ML) techniques have demonstrated potential in modeling complex clinical data; however, concerns about interpretability often limit their use.

Shapley Additive Explanations (SHAP), a popular model-agnostic tool, provides patient-level insights by measuring the contribution of each feature to a single prediction, which can be used to connect model outputs to clinically meaningful variables that can be used to display individualized risk factors [10, 11]. However, these insights at the personal level can be limited in clinical settings that require a broader understanding of population trends. Partial dependence plots (PDPs) offer an alternative method, illustrating the average effect of one or more features on predicted outcomes across the entire population, rather than focusing on isolated, individual-level attributions [6, 12]. PDPs are especially useful in clinical prediction models because they can demonstrate multidimensional and nonlinear relationships, highlight population-level trends, and show interactions between variables without removing features from their clinical context. Unlike SHAP, PDPs avoid creating data silos and offer a more comprehensive view of risk factors, which is crucial for understanding complex phenomena such as volume-outcome relationships in TJA. More importantly, the goals of clinical guideline development require an interpretability framework that aligns with population-level evidence synthesis. Frameworks such as the Grading of Recommendations Assessment, Development and Evaluation (GRADE) Working Group emphasize marginal effects across the population rather than individual-specific attributions, meaning that interpretability tools must be evaluated based on their ability to capture cohort-level risk patterns [13, 14]. PDPs inherently estimate these marginal risk relationships for the entire cohort, whereas SHAP generates individualized attribution scores that vary from patient to patient and therefore do not provide stable thresholds or population-level risk gradients needed for guideline formation. Despite this conceptual distinction, there is a lack of work evaluating how these methods perform in clinical datasets. This gap is particularly relevant in TJA, where clinicians require both individualized risk explanations and reliable population-level insights to guide optimization practices.

Therefore, this study aims to compare the interpretive usefulness of PDPs and SHAP in explaining ML-based risk prediction models for 30-day postoperative complications after TJA. Additionally, we sought to demonstrate the ability of PDPs to provide population-level insights using TJA as an outcome assessment tool, thereby enabling further understanding of ML-informed perioperative decision-making within a large, representative patient cohort.

## Methods

### Study design and data source

We conducted a retrospective cohort study using the American College of Surgeons National Surgical Quality Improvement Program (NSQIP) database. NSQIP is a prospectively collected, risk-adjusted database that records 30-day postoperative outcomes from more than 700 participating hospitals. We identified all TJA procedures in the ACS-NSQIP database from 2019–2023, yielding an initial cohort of 557,307 cases. The initial cohort included: CPT 27130 (primary THA, *n* = 210,484, 37.8%), CPT 27447 (primary TKA, *n* = 307,342, 55.2%), CPT 27134/27137/27138 (hip revisions, *n* = 15,531, 2.8%), CPT 27487 (knee revisions, *n* = 15,750, 2.8%), and CPT 27446 (unicompartmental TKA, *n* = 8,200, 1.5%). To ensure clinical homogeneity, we refined the cohort to primary TJA only, excluding all 31,281 revision cases (5.6%) and 8,200 unicompartmental procedures (1.5%). The final analytic cohort included 517,826 primary TJA cases (40.7% hip, 59.3% knee).

### Data preprocessing and quality control

Data preprocessing was carried out using STATA 17.0 (StataCorp, College Station, TX) to ensure high data quality for machine learning analysis. Age variables coded as “90+ ” (*n* = 3,325) were converted to the numeric value 90. NSQIP-specific missing value codes (−99, −1) were transformed into proper missing values, revealing missing data across several variables: hemoglobin A1c (90.2%), elective surgery status (60.7%), weight loss > 10% (60.7%), preoperative albumin (44.9%), preoperative creatinine (7.4%), and preoperative hematocrit (6.6%). We performed two approaches to assess the impact of missing data handling on model performance and conclusions: baseline median imputation and multiple imputation with five independent iterations. The baseline approach used median imputation for continuous variables. Mode imputation was not performed as the majority of categorical variables (16 of 18) had complete or near-complete data (< 5% missing), and for the two variables with substantial missingness (elective surgery status [60.7% missing] and weight loss status [60.7% missing], missing values were retained as distinct categories to preserve data integrity. For multiple imputation, we generated five independent imputed datasets using chained equations, trained separate models on each, and pooled the results. Model development prioritized the method that yielded the highest discriminative performance. No patients were excluded due to missing data to produce a complete dataset for machine learning. Physiologic range filters were applied to remove biologically implausible values: body mass index (BMI) less than 10 or greater than 80 kg/m^2^ (3,415 cases), operative time > 12 h (71 cases), hematocrit less than 15% or over 60% (322 cases), albumin less than 1 or greater than 6 g/dL (166 cases), and hemoglobin A1c less than 4% or over 20% (17 cases).

### Machine learning model development

We built binary classification models to predict a composite 30-day complication outcome, including surgical site infections, wound disruption, pneumonia, pulmonary embolism, renal failure, urinary tract infection, stroke, cardiac events, bleeding requiring transfusion, venous thromboembolism, sepsis, return to the operating room, and unplanned readmission. Twenty predictor variables were selected based on clinical relevance and data completeness: demographics (age, sex, BMI), comorbidities (diabetes, smoking status, functional status, hypertension, COPD, CHF, steroid use, weight loss, bleeding disorders), preoperative laboratory values (hematocrit, creatinine, hemoglobin A1c, albumin), and surgical factors (ASA class, operative time, elective status, procedure type). The dataset (*n* = 517,826) was split into training (80%, *n* = 414,260) and test (20%, *n* = 103,566) sets using stratified random sampling based on the 30-day complication outcome. This stratification ensured proportional representation of complicated and uncomplicated cases in both sets, resulting in identical complication rates of 6.67% in both training and test sets (absolute difference < 0.01%). Baseline characteristics for all 20 predictor variables were compared between training and test sets to verify representativeness (Supplementary Table S1); no significant differences were observed (all *P* > 0.05 after Bonferroni correction for multiple comparisons). All model training, hyperparameter optimization via fivefold cross-validation, and feature importance calculations were performed exclusively on the training set, with the test set reserved solely for final performance evaluation and interpretability analysis. Model performance was evaluated using the area under the receiver operating characteristic curve (AUC) with bootstrap confidence intervals.

### Baseline model (primary analysis)

Our primary analysis employed a Random Forest classifier with the 20 main effect predictors described above, without interaction terms. To manage computational efficiency for the large training set, hyperparameter tuning explored the following ranges: number of trees in the ensemble (100–300), maximum tree depth (5–15 levels), minimum samples required to split a node (5–15), and minimum samples required in leaf nodes (2–8) using fivefold cross-validation on a stratified subsample of 50,000 cases (12.1% of training data, maintaining 6.666% complication rate). Final models were then retrained on the complete training set (*n* = 414,260) using optimized parameters. This baseline model was used for: (1) primary comparative interpretability analysis between SHAP and partial dependence plots, (2) directional concordance validation comparing PDP trends with SHAP attributions across 5,000 test-set patients, and (3) comparative patient case examples positioning three representative patients on population risk curves.

### Interaction model (secondary analysis)

To demonstrate partial dependence plots’ capability for visualizing complementary effects between predictors, we developed a secondary Random Forest model incorporating 24 explicit interaction terms in addition to the 20 main effects (44 total features, Supplemental Table S2). Interaction terms were created as the product of standardized main effect values. The same train-test split was used, and hyperparameter optimization followed the identical procedure as the baseline model.

### Interpretability analysis methods

We implemented a two-stage interpretability approach, prioritizing features with meaningful predictive contributions. First, we calculated permutation importance using 30 bootstrap iterations, measuring relative influence through random shuffling of each feature and observing AUC decrease. Features with less than 5% relative influence were excluded to focus on clinically actionable factors and prevent confounding from low-signal variables.

Second, we created threshold-based PDPs for significant features. We generated one-dimensional plots for key variables (BMI, age, ASA class, operative time, hematocrit) and two-dimensional interaction plots for clinically meaningful pairs (BMI × Age, Diabetes × Smoking, Age × ASA class, Functional Status × Age, BMI × Diabetes, Smoking × COPD). PDPs display risk relationships across the central 90% (5th–95th percentile) of each variable’s distribution, excluding outliers. For computational efficiency during 2D PDP generation, we sampled 5,000–10,000 cases from the training dataset, preserving statistical validity while enabling comprehensive analysis of all 24 interaction pairs. All analyses used Python 3.9.

To illustrate SHAP and PDP interpretability methods, we selected three representative patient cases using objective percentile-based criteria. Patients were chosen at the 10th, 50th, and 90th percentiles of model-predicted complication risk to represent low-risk, median-risk, and high-risk patient categories across the risk spectrum. For each patient, we calculated SHAP values and extracted their position on population-level PDP curves for three continuous predictors: preoperative hematocrit, age, and operative time. For PDP visualization, we computed bootstrap confidence intervals using 50 random resamples with replacement from the training set (*n* = 2,000 patients per resample) to quantify uncertainty in the population risk curves. Each patient’s overall predicted risk integrates contributions from all 20 model predictors, while their position on an individual PDP curve reflects the population-average risk at that specific feature value. We created side-by-side visualizations with SHAP attributions showing individual contributions in log-odds units, and PDP curves showing population risk patterns.

### Statistical validation of PDP-SHAP directional concordance

To validate that PDP-derived clinical thresholds reflect genuine model relationships rather than visualization artifacts, we performed a direction agreement analysis comparing PDP trends with SHAP attributions. For each of the 20 predictors, we: (1) determined the overall PDP trend direction by calculating whether risk increased or decreased across the feature’s range (positive or negative slope), (2) calculated SHAP values for 5,000 randomly sampled test-set patients, (3) computed the Spearman correlation between each feature’s values and its corresponding SHAP attributions across patients, and (4) assessed directional agreement by comparing the sign of the PDP trend with the sign of the SHAP-feature correlation. Agreement was calculated both unweighted (simple proportion of features with matching directions) and weighted by each feature’s relative importance from permutation analysis, giving greater emphasis to clinically influential predictors. Statistical significance of SHAP-feature correlations was assessed using two-tailed tests with Bonferroni correction for multiple comparisons (α = 0.05/20 = 0.0025).

## Results

### Study population and baseline characteristics

The final analytical cohort consisted of 517,826 TJA cases, with TKA accounting for 307,342 (59.4%) compared to THA (210,484, 40.7%). The average age of TJA patients was 67.0 ± 10.0 years, and female patients accounted for 301,949 (58.3%) of the procedures. The mean BMI was 31.8 ± 6.5 kg/m^2^. ASA classifications showed 1.9% ASA 1, 46.8% ASA 2, 49.2% ASA 3, and 2.0% ASA 4 patients. Common comorbidities included hypertension in 310,444 (60.0%) patients, diabetes mellitus in 84,707 (16.4%), current smoking status in 44,053 (8.5%), chronic steroid use in 20,756 (4.0%), chronic obstructive pulmonary disease in 17,735 (3.4%), congestive heart failure in 9,481 (1.8%), and bleeding disorders in 9,991 (1.9%) patients. Preoperative laboratory values were generally within normal ranges, including a mean hematocrit of 41.4 ± 4.1%, albumin 4.2 ± 0.4 g/dL, creatinine of 0.9 ± 0.4 mg/dL, and hemoglobin A1c 5.9 ± 0.9%. The average operative time was 90.9 ± 35.6 min. Among the 203,554 patients with documented acuity status, 199,212 (97.9%) procedures were performed electively (Table 1).

**Table 1:** Baseline characteristics of included patients

Characteristic	Total Cohort (*N* = 517,826)
Age (years)	67.0 ± 10.0
Female Sex (*N*, %)	301,949 (58.3%)
BMI (kg/m^2^)	31.8 ± 6.5
**Procedure Type (*N*, %)**	
Total Hip Arthroplasty	210,484 (40.7%)
Total Knee Arthroplasty	307,342 (59.4%)
**ASA Classification (*N*, %)**	
ASA 1	9,568 (1.9%)
ASA 2	242,181 (46.8%)
ASA 3	254,926 (49.2%)
ASA 4	10,424 (2.0%)
ASA 5	34 (0.0%)
**Comorbidities (*N*, %)**	
Diabetes Mellitus	84,707 (16.4%)
Current Smoking	44,053 (8.5%)
Hypertension	310,444 (60.0%)
COPD	17,735 (3.4%)
Congestive Heart Failure	9,481 (1.8%)
Chronic Steroid Use	20,756 (4.0%)
Weight Loss > 10%	314 (0.1%)
Bleeding Disorders	9,991 (1.9%)
Independent Functional Status	507,211 (98.0%)
**Preoperative Laboratory Values**	
Hematocrit (%)	41.4 ± 4.1
Albumin (g/dL)	4.2 ± 0.4
Creatinine (mg/dL)	0.9 ± 0.4
Hemoglobin A1c (%)	5.9 ± 0.9
**Surgical Variables**	
Operative Time (minutes)	90.9 ± 35.6
Elective Surgery (*N*, %)*	199,212 (97.9%)
**Outcomes**	
30-Day Complications (*N*, %)	34,694 (6.7%)

^*^Acuity status: Elective vs non-elective percentages are calculated among the 203,554 patients with documented acuity status; 199,212 (97.9%) of these procedures were elective

### Feature importance and baseline model performance

**Fig. 1:**
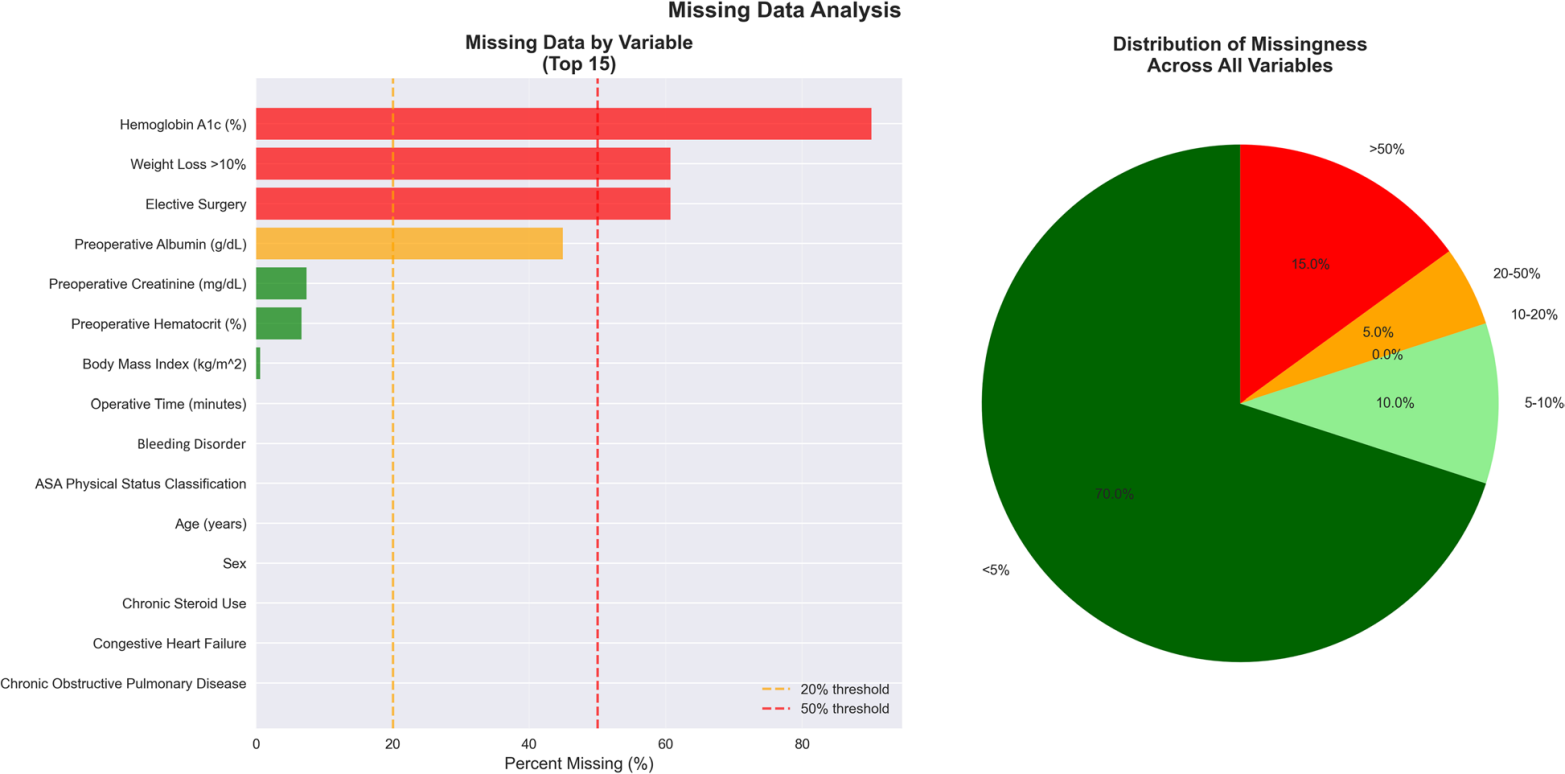
Missing data patterns for the top 15 features used in machine learning modeling. Bar chart illustrating the percentage of missing values for the 15 predictors with the highest missingness, ranked from highest to lowest. Color coding reflects severity: red indicates greater than 50 percent missingness, yellow indicates 20–50 percent, and green indicates less than 20 percent. A summary pie chart depicting the overall distribution of missing data across all predictors is also included

Four features possessed missing data exceeding 40%: Hemoglobin A1c (90.2% missing, importance rank 13/20), Elective Surgery (60.7% missing, rank 14/20), Weight Loss > 10% (60.7% missing, rank 20/20), and Preoperative Albumin (44.9% missing, rank 5/20, Fig. 1). To isolate the effect of imputation methodology, we compared two approaches using Random Forest models with default hyperparameters. Baseline median imputation achieved test AUC = 0.6492. Multiple imputation across five iterations produced a mean AUC = 0.6440 ± 0.0013 (range: 0.6420 to 0.6453), representing a negligible difference of 0.0052 AUC points. The low standard deviation across imputations indicates stable model behavior regardless of imputation strategy, validating our use of median imputation for the final optimized model. Our final hyperparameter-optimized Random Forest model with median imputation achieved test AUC = 0.6777 with all 20 features. A reduced model excluding the four high-missingness features achieved AUC = 0.6484, demonstrating that retention of these imputed variables improved discrimination (ΔAUC = 0.0293). Both the Random Forest and Gradient Boosting models, utilizing 20 primary clinical features, demonstrated moderate predictive performance. Random Forest achieved a training AUC = 0.703 and a testing AUC = 0.678, while Gradient Boosting reached a training AUC = 0.689 and a testing AUC = 0.681. When 24 explicit interaction terms were incorporated into the models, training performance improved: Random Forest posted a training AUC = 0.754 and a testing AUC = 0.668, whereas Gradient Boosting showed a training AUC = 0.763 and a testing AUC = 0.668.

**Fig. 2:**
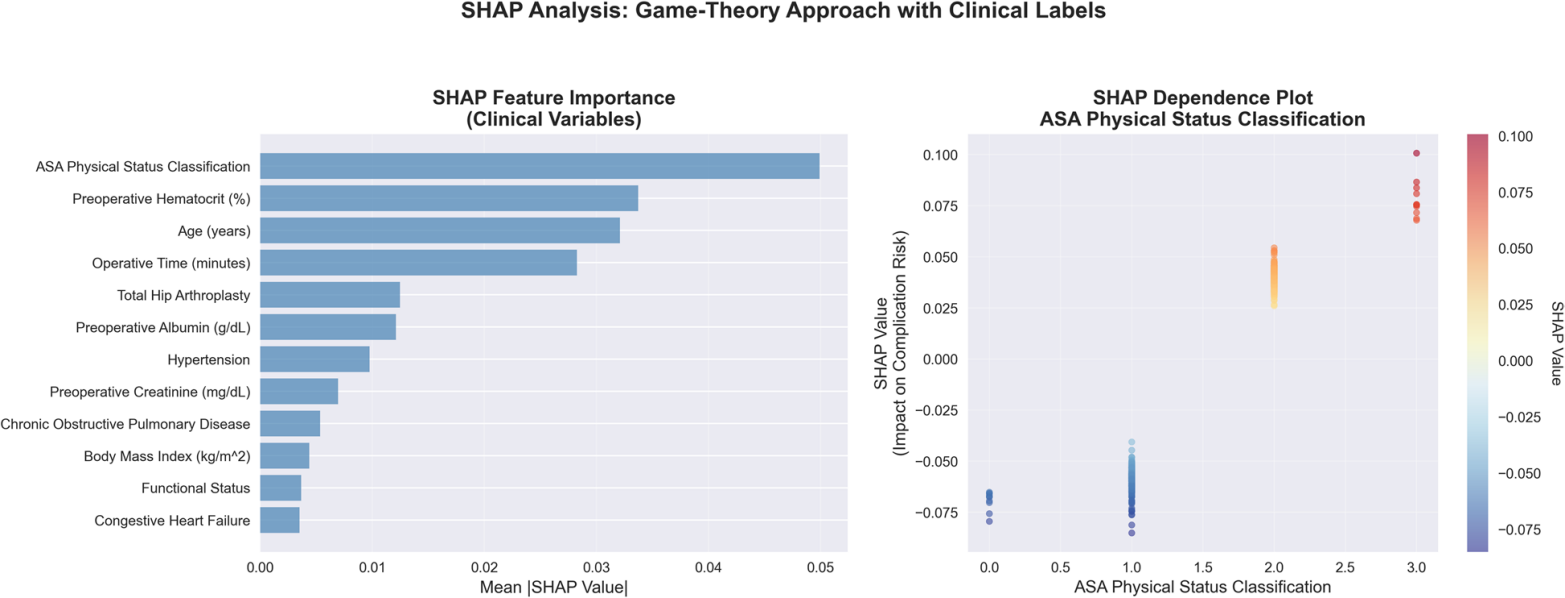
SHAP feature importance analysis. SHAP feature importance rankings and dependence plot for the most influential variable (ASA Classification), showing individual-level attributions across 500 randomly sampled cases using the baseline Random Forest model

SHAP analysis of the baseline Random Forest model identified the importance of various features for individual patient predictions. The ASA Physical Status Classification was the most influential predictor (SHAP importance 0.050), followed by preoperative hematocrit (0.034), age (0.032), operative time (0.028), and procedure type (THA vs TKA, 0.012). Lower-ranked factors included preoperative albumin (0.012), hypertension (0.010), and preoperative creatinine (0.007) (Fig. 2).

### Initial partial dependence analysis

**Fig. 3:**
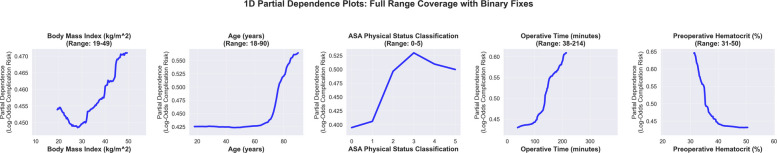
Initial partial dependence analysis. One-dimensional partial dependence plots for key clinical variables using the baseline Random Forest model (20 main features), showing dose–response relationships across the full variable spectrum in partial dependence units

Initial 1D PDPs were generated using the baseline Random Forest model (with 20 main features only) to establish comprehensive dose–response relationships across all clinical variables. Before calculating variable influence, 1D PDPs were created from the baseline models for key clinical features that reveal dose–response relationships. Age showed a steady increase in risk, from 0.424 to 0.564 partial dependence units, between ages 18 and 90, with a clear inflection point at age 70, where the risk acceleration became significantly more noticeable. ASA classification demonstrated risk stratification across classes (0.395 to 0.530 partial dependence units). Operative time showed a risk increase from 0.430 to 0.609 partial dependence units (Fig. 3).

Proper handling of six binary variables (without interpolation) revealed risk differences. Chronic Obstructive Pulmonary Disease showed the most significant effect (+0.222 log-odds difference), followed by Congestive Heart Failure (+0.177 log-odds), bleeding disorder (+0.091 log-odds), THA (+0.085 log-odds), Hypertension (+0.071 log-odds), chronic steroid use (+0.033 log-odds), and current smoking (+0.032 log-odds).

### Directionality agreement between SHAP and PDP

Direction agreement analysis across 5,000 test-set patients revealed high concordance between PDP trends and SHAP attributions (Table 2). Of 20 predictors, 18 (90.0%) showed matching directional effects between PDP slope and SHAP-feature correlation. When weighted by permutation importance, agreement increased to 97.8%. The two features with discordant directions (Functional Status and Elective Surgery) had low importance rankings (importance < 0.01). All SHAP-feature correlations for concordant predictors were statistically significant (*P* < 0.0001 after Bonferroni correction). High-importance predictors demonstrated strong correlation magnitudes: ASA classification (ρ = +0.879, *P* < 0.0001), operative time (ρ = +0.838, *P* < 0.0001), preoperative hematocrit (ρ = −0.862, *P* < 0.0001), and age (ρ = +0.905, *P* < 0.0001).

**Table 2:** Convergent validity of feature effects: Directional concordance between partial dependence plots and SHAP attributions

Feature	PDP Effect Direction	SHAP Correlation (ρ)	Directionality Agreement	*P*-value	SHAP Importance Rank
ASA Physical Status Classification	Higher class → Higher risk (+)	+ 0.879	Yes	< 0.0001	1
Preoperative Hematocrit (%)	Higher value → Lower risk (−)	− 0.862	Yes	< 0.0001	2
Age (years)	Older age → Higher risk (+)	+ 0.905	Yes	< 0.0001	3
Operative Time (minutes)	Longer time → Higher risk (+)	+ 0.838	Yes	< 0.0001	4
Total Hip Arthroplasty	THA vs TKA → Higher risk (+)	+ 0.852	Yes	< 0.0001	5
Preoperative Albumin (g/dL)	Higher value → Lower risk (−)	− 0.874	Yes	< 0.0001	6
Hypertension	Present → Higher risk (+)	+ 0.850	Yes	< 0.0001	7
Preoperative Creatinine (mg/dL)	Higher value → Higher risk (+)	+ 0.470	Yes	< 0.0001	8
Sex	Female vs Male → Lower risk (−)	− 0.845	Yes	< 0.0001	9
Weight Loss > 10%	Present → Lower risk (−)	− 0.827	Yes	< 0.0001	10
Elective Surgery	Elective → Lower risk (−)	+ 0.645	No	< 0.0001	11
Body Mass Index (kg/m^2^)	Higher BMI → Higher risk (+)	+ 0.472	Yes	< 0.0001	12
Current Smoking	Present → Higher risk (+)	+ 0.468	Yes	< 0.0001	13
Diabetes Mellitus	More severe → Lower risk (−)	− 0.405	Yes	< 0.0001	14
Chronic Steroid Use	Present → Higher risk (+)	+ 0.327	Yes	< 0.0001	15
COPD	Present → Higher risk (+)	+ 0.304	Yes	< 0.0001	16
Congestive Heart Failure	Present → Higher risk (+)	+ 0.257	Yes	< 0.0001	17
Bleeding Disorder	Present → Higher risk (+)	+ 0.256	Yes	< 0.0001	18
Functional Status	More dependent → Lower risk (−)	+ 0.229	No	< 0.0001	19
Hemoglobin A1c (%)	Higher value → Lower risk (−)	− 0.048	Yes	0.001	20

Convergent validity across PDP and SHAP applied to the base Random Forest model (test AUC = 0.6777, *n* = 103,566 patients). All analyses evaluated the relationship between predictor variables and the risk of 30-day postoperative complications. PDP Effect Direction describes the relationship between increasing feature values and complication probability observed in PDPs, with continuous variables indicating the direction of change (e.g., “Higher value → Lower risk”) and binary/categorical variables indicating the comparison groups (e.g., “Present → Higher risk”). SHAP Correlation (ρ) represents the Spearman rank correlation coefficient between each feature's values and its corresponding SHAP attributions calculated across 5,000 randomly sampled test-set patients, measuring whether higher feature values are associated with positive (risk-increasing) or negative (risk-protective) SHAP attributions. An agreement indicates directional concordance. P-value reports the statistical significance of the SHAP-feature correlation using two-tailed tests with Bonferroni correction for 20 comparisons (corrected α = 0.0025). SHAP Importance Rank indicates relative feature importance based on mean absolute SHAP values, where lower ranks denote greater influence on model predictions

### Threshold-based feature selection for population-level analysis

**Fig. 4:**
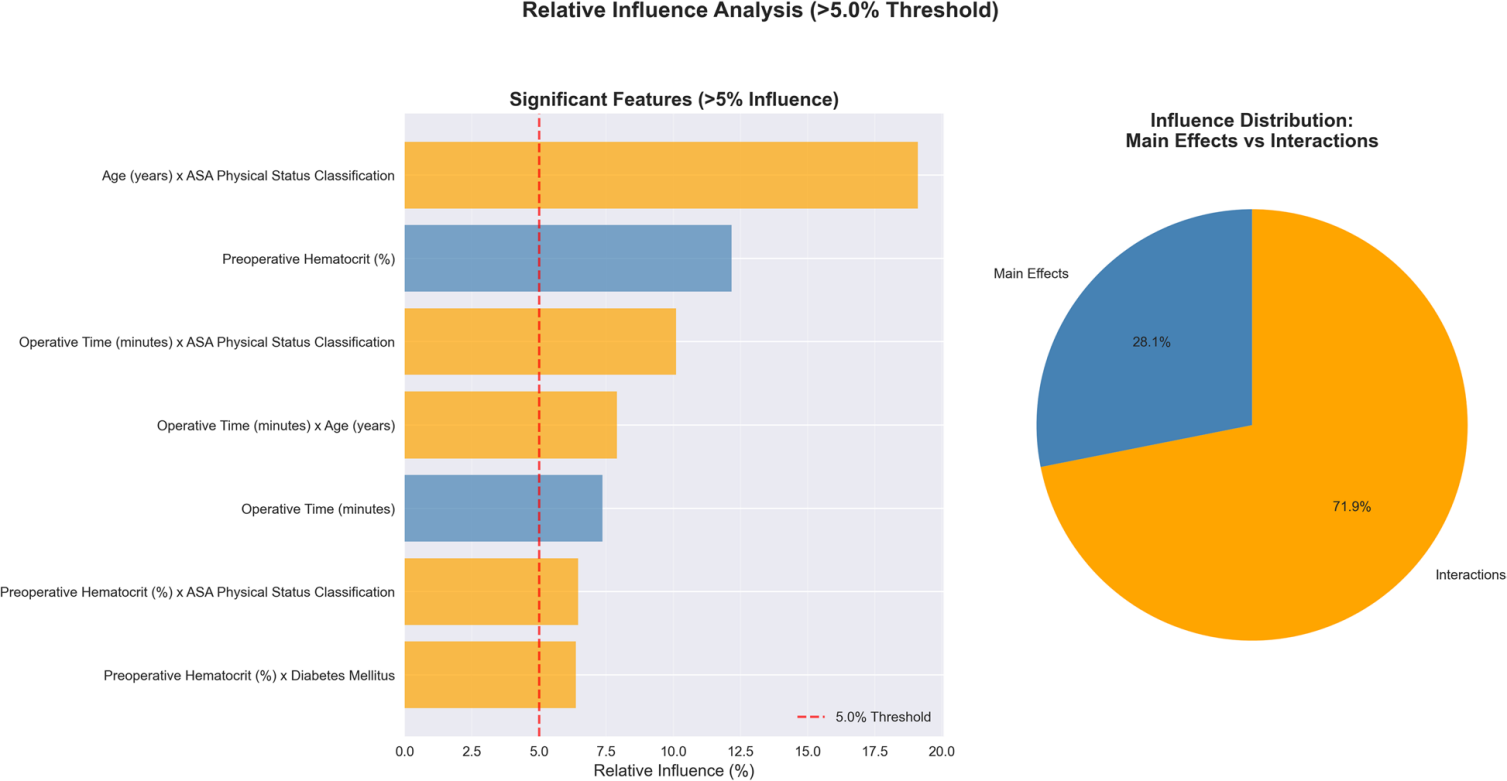
Permutation importance analysis: features exceeding 5% relative influence threshold. The left panel displays the seven features exceeding the 5% relative influence threshold based on permutation importance analysis with 30 repetitions on the test set (*n* = 103,566). Features are color-coded: blue bars represent main effects while orange bars represent interaction terms. The red dashed vertical line marks the 5% threshold for inclusion. The right panel shows the relative distribution of influence within these seven significant features: main effects contribute 28.1% (19.5% of total model influence), while interaction effects contribute 71.9% (49.9% of total model influence). Together, these seven features account for 69.5% of total model predictive power, with the remaining 30.5% distributed among 37 features below the 5% threshold

To focus on features with clinically meaningful and statistically robust contributions, we used a 5% relative influence threshold, calculated using permutation importance across 30 bootstrap iterations. This approach prioritized features with enough signal strength to support reliable clinical decisions while avoiding spurious pattern detection in low-influence variables. Out of 44 total features, 7 exceeded the 5% relative influence threshold: age × ASA classification (19.10% ± 0.141%), operative time × ASA classification (10.10% ± 0.073%), operative time × age (7.90% ± 0.043%), preoperative hematocrit × ASA (6.45% ± 0.062%), preoperative hematocrit × diabetes (6.37% ± 0.056%), operative time (7.37% ± 0.036%), and preoperative hematocrit (12.16% ± 0.056%) (Fig. 4). These significant features were chosen for detailed partial dependence analysis.

### Threshold-based partial dependence analysis: Population-level clinical insights

**Fig. 5:**
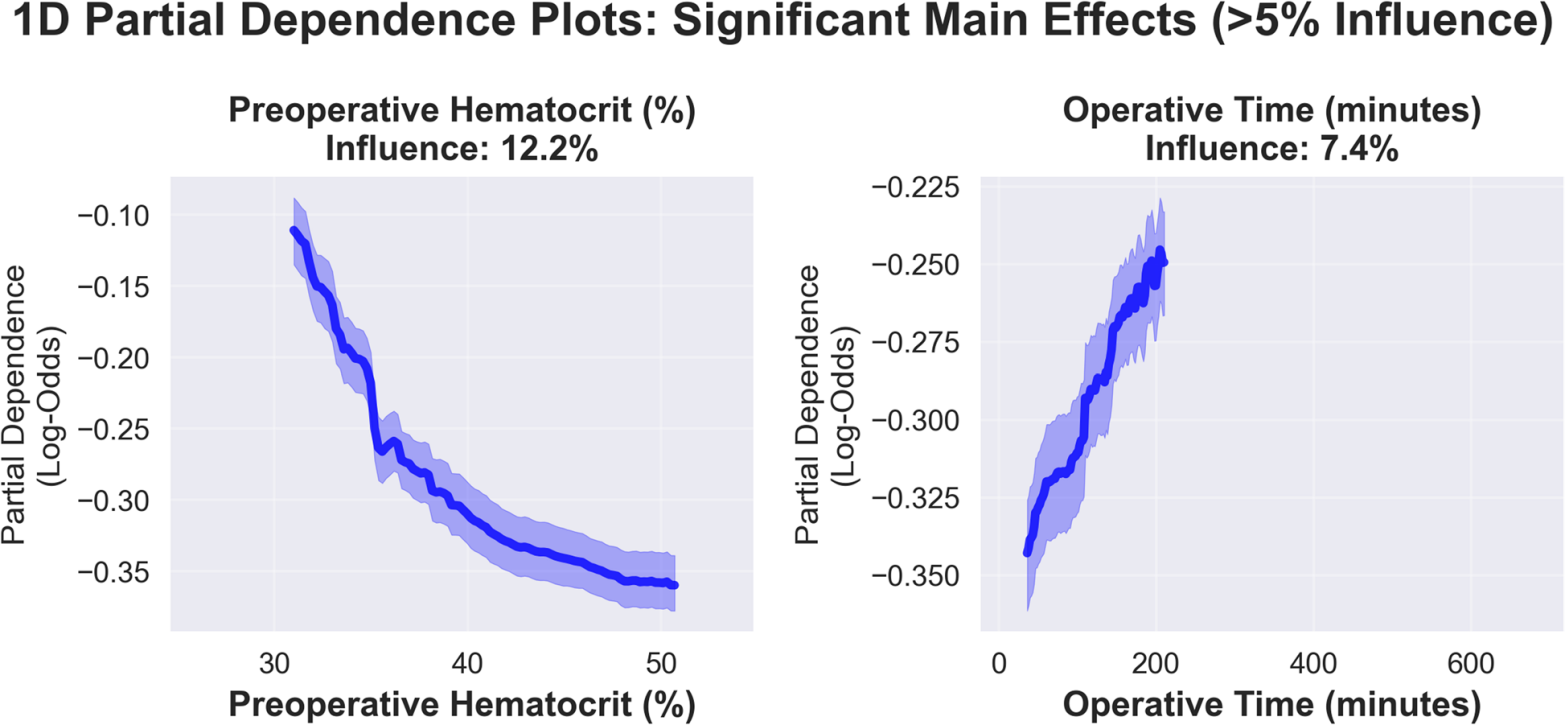
Threshold-based partial dependence analysis. One-dimensional partial dependence plots for statistically significant features (> 5% relative influence) using the interaction Random Forest model, showing population-level risk patterns in log-odds with confidence intervals

PDPs meeting significance criteria (defined as contributing more than 5% influence on model performance) were generated using the interaction Random Forest model (20 main features and 24 interaction terms) to capture the full complexity of feature relationships. Operative time exhibited a near-linear increase in risk, with the risk gradually rising from −0.343 to −0.245 log odds. Preoperative hematocrit had a protective effect, with risk decreasing from −0.111 log-odds at 30% to −0.360 at 50% (Fig. 5).

### Interaction analysis: complementary risk identification

**Fig. 6:**
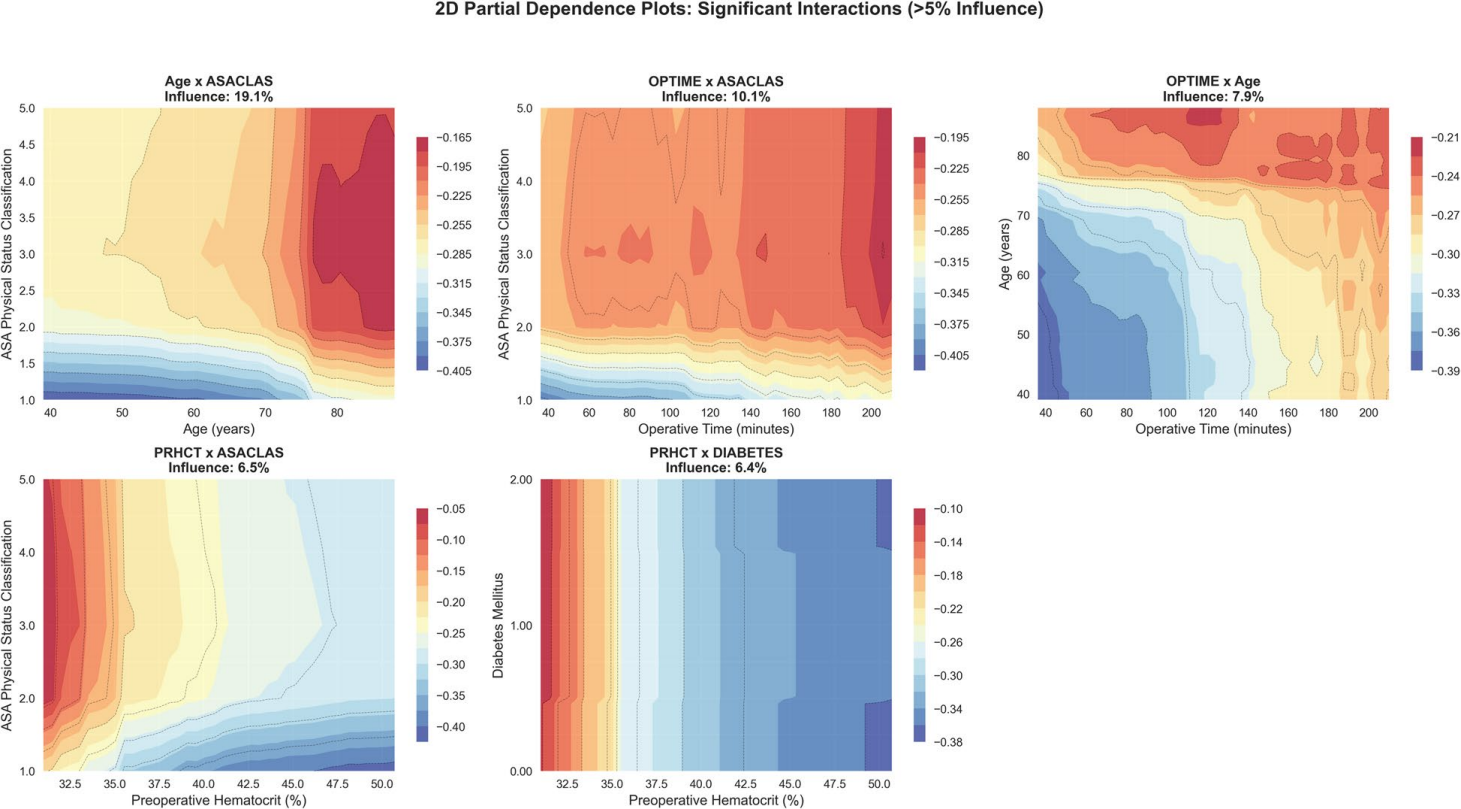
Two-dimensional interaction analysis. Two-dimensional partial dependence plots for significant feature interactions exceeding the 5% influence threshold, revealing complementary risk combinations and high-risk zones for targeted clinical interventions. Diabetes labeling: 2 = insulin dependent, 1 = non-insulin dependent, and 0 = none

Among features exceeding the 5% relative influence threshold, interaction effects contributed 49.9% of model influence compared to 19.5% from main effects, together accounting for 69.5% of total model predictive power. Within these seven most influential features, interaction effects dominated (71.9%) over main effects (28.1%), with the five most significant interaction pairs being age × ASA, operative time × ASA, preoperative hematocrit × ASA, preoperative hematocrit × diabetes, and operative time × age. The age × ASA classification interaction (19.1% relative importance) showed risk ranging from −0.402 to −0.171 log-odds, with the highest-risk combinations occurring in elderly patients with moderate systemic disease (Age 86.7 years + ASA 3: −0.171 log-odds). The operative time × ASA classification interaction (10.1% relative importance) demonstrated risk spanning −0.405 to −0.199 log-odds, with prolonged procedures in ASA 3 patients reaching peak risk (OPTIME 205.5 min + ASA 3: −0.199 log-odds). The preoperative hematocrit × diabetes interaction (6.4% relative importance) exhibited a risk span of −0.362 to −0.104 log-odds, showing that anemia in diabetic patients significantly amplifies risk. The operative time × age interaction (7.9% relative importance, −0.390 to −0.222 log-odds) revealed that elderly patients undergoing prolonged procedures face compounded risk (OPTIME 116.3 min + Age 86.7 years: −0.222 log-odds). Preoperative hematocrit × ASA interaction (6.5% relative importance, −0.414 to −0.058 log-odds) demonstrated that the highest-risk combinations occurred in anemic patients with higher ASA class (hematocrit 31.0% and ASA 3: −0.058 log-odds) (Fig. 6).

### Case-based comparison of SHAP vs. PDP interpretability

**Fig. 7:**
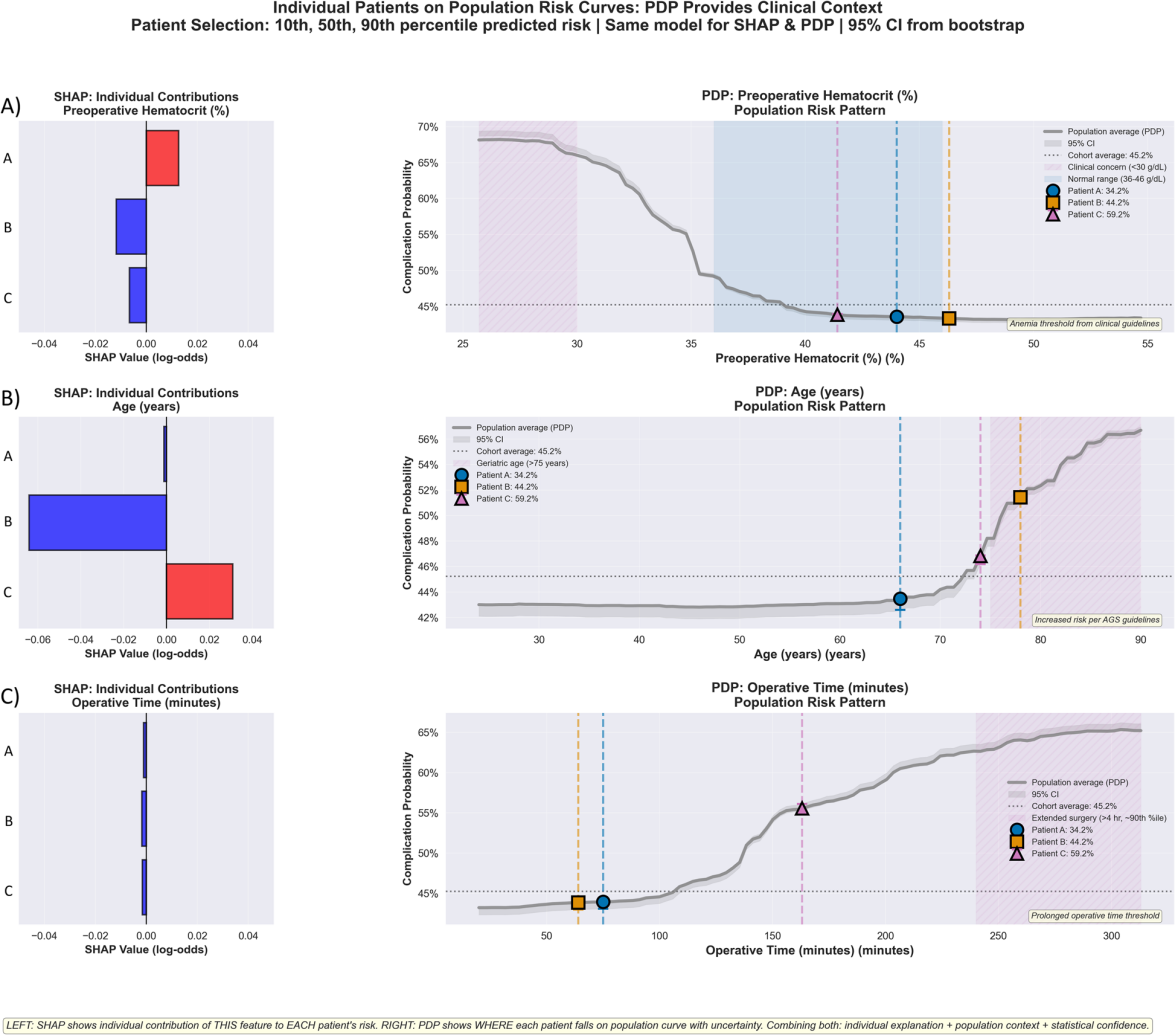
Individual patients positioned on population risk curves: comparative demonstration of SHAP individual attributions versus PDP. Side-by-side comparison of SHAP (left panels) and partial dependence plots (right panels) for three representative patients selected at the 10th, 50th, and 90th percentiles of uncalibrated predicted risk scores (relative risk rankings of 34.2%, 44.2%, and 59.2% from Random Forest output): Patient A (low-risk, 34.2%), Patient B (median-risk, 44.2%), and Patient C (high-risk, 59.2%), with complete characteristics in Table 3. Left panels show SHAP attributions quantifying individual feature contributions in log-odds units. Right panels show population PDP curves with 95% confidence intervals from bootstrap resampling (50 iterations), with patients positioned using color-coded symbols (circle = Patient A, square = Patient B, triangle = Patient C). Rows display (**A**) preoperative hematocrit, (**B**) age, and (**C**) operative time. For hematocrit, Patient C’s value of 41.4% shows a modest SHAP contribution (+0.0126), but PDP positions are near the < 38% risk acceleration threshold, identifying an optimization opportunity that is invisible in SHAP alone. For age, Patients B and C show divergent SHAP values (+0.0309 vs −0.0012), yet PDP positions both in the geriatric risk zone (> 75 years), warranting systematic protocols. For operative time, all patients show near-zero SHAP values (−0.0015 to −0.0011) despite Patient C’s 163-min procedure substantially exceeding the 120-min population threshold revealed by PDP (risk increases 45% to 58% at 240 min). This comparison demonstrates complementary roles: SHAP for individualized patient counseling, PDP for institutional guideline development through population thresholds

Three patients were selected at the 10th, 50th, and 90th percentiles of predicted complication risk and plotted along both SHAP attribution values and corresponding population-level PDP curves: Patient A (34.2%), Patient B (44.2%), and Patient C (59.2%, Fig. 7). Patient characteristics are provided in Table 3. Patient A had hematocrit 44.0%, age 66 years, and operative time 75 min; Patient B had hematocrit 46.3%, age 78 years, and operative time 64 min; and Patient C had hematocrit 41.4%, age 74 years, and operative time 163 min. For preoperative hematocrit (Fig. 7A), SHAP values were − 0.0066 (Patient A), −0.0118 (Patient B), and + 0.0126 (Patient C). The partial dependence plot showed a gradual increase in population-level risk as hematocrit decreased, with a steeper slope below approximately 38%. Patient C’s hematocrit value (41.4%) aligned with the portion of the PDP curve where risk begins to rise relative to higher hematocrit ranges (> 42%). For age (Fig. 7B), SHAP values were + 0.0309 (Patient A), −0.0641 (Patient B), and −0.0012 (Patient C). The PDP demonstrated an approximately linear increase in population risk from age 50 to 85, with higher values above 75 years. Both Patient B (78 years) and Patient C (74 years) fell within the upper segment of this distribution. For operative time (Fig. 7C), SHAP values were −0.0015 (Patient A), −0.0018 (Patient B), and −0.0011 (Patient C). The PDP indicated a relatively flat region below 120 min, followed by increasing population-level risk with longer operative durations, reaching its upper range near 240 min. Patient C (163 min) fell above the inflection zone, whereas Patients A and B remained within the lower-duration region.

**Table 3:** Baseline characteristics of representative patient archetypes

Variable	Patient A (Low-Risk, 10th %ile)	Patient B (Median-Risk, 50th %ile)	Patient C (High-Risk, 90th %ile)
Overall Predicted Risk	34.2%	44.2%	59.2%
Age (years)	66	78	74
Sex	Male	Female	Male
Body Mass Index (kg/m^2^)	33.8	23.0	22.7
Diabetes Mellitus	Non-insulin Dependent	Non-insulin Dependent	Insulin Dependent
Current Smoking	No	No	No
Functional Status	Independent	Independent	Independent
Hypertension	Yes	Yes	No
COPD	No	No	No
Congestive Heart Failure	No	No	No
Chronic Steroid Use	No	No	No
Weight Loss > 10%	Yes	Yes	No
Preoperative Hematocrit (%)	44.0	46.3	41.4
Preoperative Albumin (g/dL)	4.2	4.2	4.2
Preoperative Creatinine (mg/dL)	0.9	0.9	0.9
Hemoglobin A1c (%)	5.7	5.7	5.7
ASA Physical Status	Class I	Class I	Class II
Operative Time (minutes)	75	64	163
Elective Surgery	Yes	Yes	Non-elective
Procedure Type	TKA	THA	THA
Actual Outcome	No complication	No complication	No complication

### Multicollinearity assessment and method comparison

**Fig. 8:**
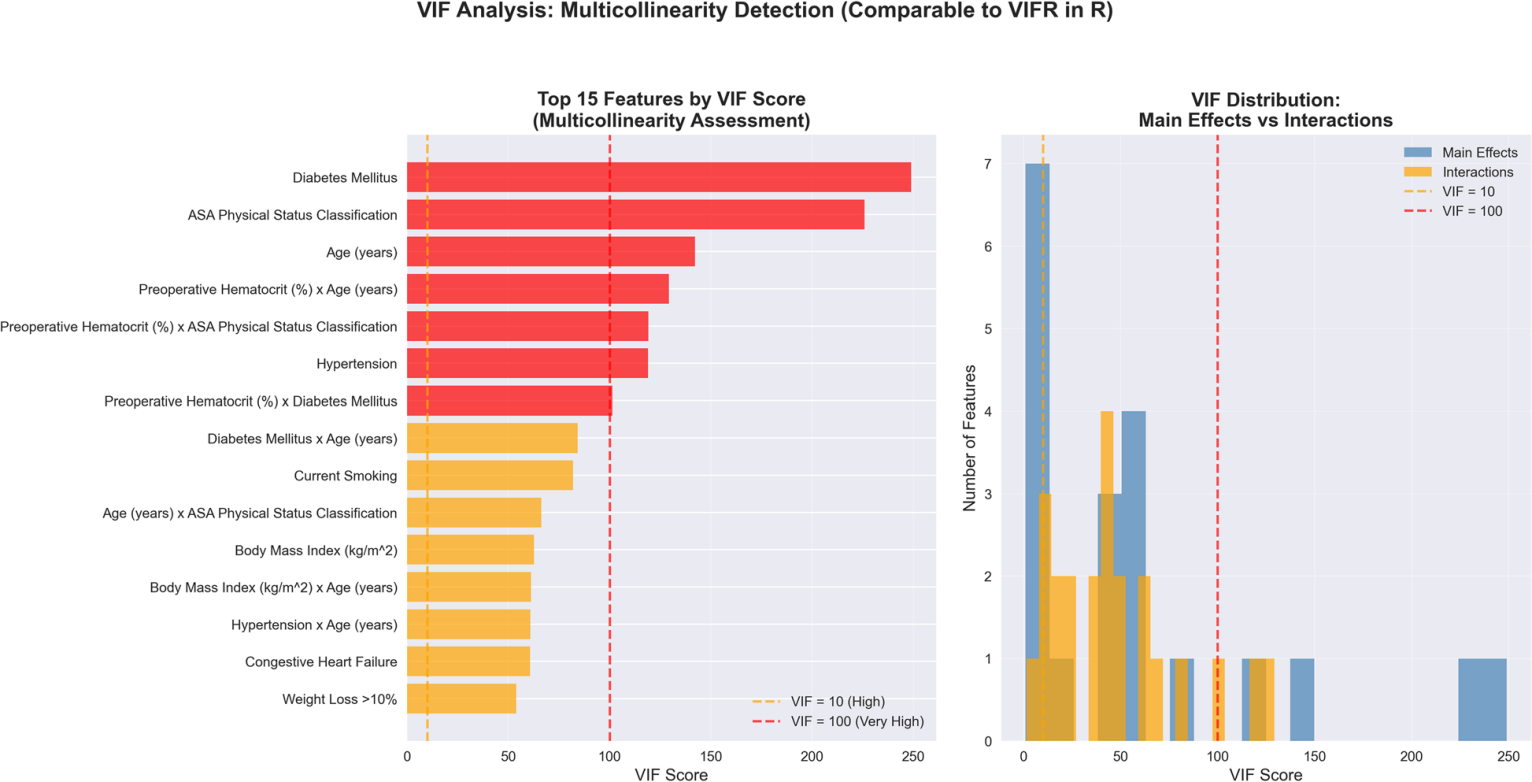
Variance inflation factor analysis. VIF analysis of main effects and interaction terms showing multicollinearity assessment

Variance Inflation Factor (VIF) analysis revealed the expected multicollinearity with interaction terms included. Main effects had a median VIF of 46.97, while interactions had a median VIF of 43.60 (overall median VIF = 45.30). The highest VIFs were for diabetes (248.9), ASA classification (225.9), and age (142.1) (Fig. 8). These high values are typical and acceptable when modeling interaction terms, as they reflect the mathematical relationship between main effects and their products rather than problematic collinearity.

## Discussion

Using NSQIP-derived TJA data, this study demonstrates that PDPs are methodologically more suitable than SHAP for population-level interpretability, particularly when clinical guideline development requires identifying stable marginal effects and risk thresholds across the cohort. By grounding our comparison in established population-evidence frameworks such as GRADE, we show that PDPs more effectively reveal the population risk gradients needed for perioperative decision-making, whereas SHAP offers individualized attributions better suited for patient-specific explanations. By analyzing 517,826 TJA procedures, we show that PDPs provide population-level interpretability that is better aligned with the goals of guideline development than the individualized attributions produced by SHAP. Among the seven features exceeding the 5% relative influence threshold, interaction effects accounted for 71.9% (contributing 49.9% of total model predictive power), while main effects contributed only 28.1% (19.5% of total model influence). This interaction dominance demonstrates that the most clinically important risk relationships are complementary rather than additive, and therefore require interpretability methods capable of visualizing multivariable effects. Partial dependence plots excel at this task through 2D visualizations that reveal how risk varies across combinations of predictors, whereas SHAP’s univariate attribution framework cannot effectively capture these population-level interaction patterns for guideline development. By grounding our comparison in established population-evidence frameworks such as GRADE, we show that PDPs more effectively reveal the population risk gradients and thresholds needed for perioperative decision-making, whereas SHAP offers individualized attributions better suited for patient-specific explanations.

Our comparative analysis clarified how PDPs and SHAP address different interpretability needs within clinical prediction. SHAP identified ASA status as the most influential contributor to individual patient risk (importance 0.050), but because it quantifies attribution at the patient level, it does not directly reveal population-wide thresholds needed for protocol development. PDPs, in contrast, characterized dose–response relationships across the full cohort and identified points where population-level risk begins to accelerate, such as preoperative hematocrit values below 38% or operative times exceeding 120 min, with total risk variation of 0.249 log-odds across the hematocrit range and 0.098 log-odds across the operative time range. By summarizing marginal effects across the cohort rather than individualized attributions, PDPs reveal the stable population thresholds that SHAP is not designed to provide. These conceptual differences are further illustrated in our comparative patient analysis.

Three representative patients at the 10th, 50th, and 90th percentiles of predicted risk showed that SHAP quantifies individual feature contributions while PDP reveals the population-level thresholds necessary for protocol development. The operative time example most clearly illustrated this distinction: Patient C’s 163-min procedure produced a near-zero SHAP value (−0.0011), which might suggest that duration was not a concerning factor for this individual, yet the PDP showed that this value lies substantially above the population threshold of 120 min, where risk increases systematically. This contrast demonstrates that SHAP explains why a patient has their predicted risk, while PDP identifies the thresholds that guide institutional protocols. A similar pattern occurred for hematocrit: Patient C’s value of 41.4% generated only a modest SHAP attribution but fell near the PDP risk-acceleration zone, highlighting an optimization opportunity that individualized importance scores alone would not detect. These case examples illustrate how PDPs align more closely with the population-level perspective required for guideline development, whereas SHAP remains most informative for individualized risk communication.

Most importantly, our 2D PDP analysis revealed multiple complementary interaction patterns that are more readily visualized with PDPs than with SHAP, particularly when assessing population-level trends. The age × ASA classification interaction (19.10% ± 0.141% relative influence) showed steep increases in modeled risk among elderly patients with higher ASA classifications. Meanwhile, preoperative hematocrit × diabetes interactions (6.37% ± 0.056%) suggested higher modeled risk among anemic diabetic patients, indicating a population pattern that may warrant further investigation in perioperative studies. Additionally, operative time × ASA classification interactions (10.10% ± 0.073%) identified higher modeled complication risk among higher-ASA patients undergoing longer procedures, reflecting a population-level association between operative duration and risk burden. Furthermore, the operative time × age interaction (7.90% ± 0.043%) showed elevated modeled risk estimates for older patients with prolonged operative times. These population-level associations may offer preliminary signals that could support future efforts in protocol development pending further validation, illustrating how PDP-derived interaction surfaces can help generate hypotheses for targeted perioperative strategies at the population level, while individualized SHAP attributions remain more appropriate for case-specific explanation and patient counseling.

The demonstrated advantages of PDPs for population-level clinical applications stem from fundamental theoretical differences in their analytical frameworks that align differently with evidence-based medicine principles. Christoph Molnar’s comprehensive theoretical framework demonstrates that PDPs illustrate average modeled effects across populations, making them suitable for clinical guidelines that require understanding of marginal effects and population distributions [15]. This population-level focus directly aligns with established clinical guideline development methods, particularly the GRADE Working Group framework, which is the global standard for evidence synthesis in healthcare [13, 14]. The GRADE methodology emphasizes population-level effect estimation over individual-level predictions when developing clinical recommendations, creating a conceptual connection between PDP’s marginal-effect perspective and the types of evidence typically used when forming clinical recommendations [16]. Conversely, SHAP’s game-theoretic approach, while mathematically impressive for individual feature attribution, is oriented toward explaining prediction differences at the single-patient level rather than estimating cohort-wide marginal effects that inform guideline development [17]. Moreover, research shows that PDP and SHAP dependence plots can diverge when interaction effects occur, with PDPs reflecting expected outputs under controlled interventions of single parameters, which may be more useful when examining potential intervention targets at the population level [18]. Regulatory frameworks also recognize the importance of interpretability and transparency for clinical decision support systems in healthcare, supporting the need to align interpretability tools with their intended use cases [19]. This theoretical foundation is supplemented by literature revealing multiple fundamental reliability issues with the SHAP methodology, which are especially problematic for clinical use. Molnar’s research demonstrates that KernelSHAP suffers from similar problems as other permutation-based interpretation methods, with model estimations potentially assigning weight to unlikely instances and producing unreliable results when sampling from marginal distributions that overlook feature dependencies [15]. Additional research indicates that SHAP can produce inconsistent results across similar models, diverging from true Shapley values even with minimal feature correlations and sometimes providing opposite signs for identical features [20]. Further studies document computational expense limitations, noting that the high computational costs of SHAP make it impractical for large-scale clinical datasets [21]. Empirical evidence also indicates that if a model is overfit, SHAP may incorrectly highlight noise variables as important, underscoring the need for careful model calibration [22]. These considerations do not diminish SHAP’s value for individualized explanation, but they highlight the importance of interpreting SHAP within the context of its assumptions and computational properties. Accordingly, PDPs contribute a complementary population-level view by marginalizing over the observed data distribution, offering stable marginal risk patterns that are more suitable for informing broad clinical guideline development [15].

This analysis has limitations that warrant acknowledgment. While our dataset includes multi-institutional NSQIP data from hundreds of hospitals, which improves generalizability compared to single-center studies, the retrospective observational design limits our ability to establish causality between identified risk factors and outcomes. Although our models achieved moderate predictive performance, the inherent complexity of machine learning models means that both PDP and SHAP interpretations are post-hoc explanations that may not fully capture the true causal relationships. Additionally, the elevated VIF values observed in our interaction models, although expected and acceptable when modeling interaction terms, reflect the mathematical relationships between main effects and their products, requiring careful consideration when developing clinical protocols. A further limitation is that the interaction-augmented models demonstrated overfitting, with gains in training AUC that did not translate to improved test performance; these models were used solely to illustrate complementary effects rather than as deployable clinical tools. Regularization strategies such as early stopping or interaction pruning were intentionally not applied, as they would reduce the interaction magnitudes that PDPs are designed to visualize. Additionally, because our study focused on comparing interpretability methods rather than maximizing predictive accuracy, the baseline test AUC of 0.678 used in the development of the PDP-derived thresholds presented should be interpreted as proof-of-concept demonstrations of population-level interpretability, with future work needed to pair these methods with fully optimized, externally validated prediction models before clinical adoption. In addition, PDPs are inherently limited because they estimate average marginal effects and cannot account for individualized prediction differences, which limits their use for patient-specific counseling. SHAP provides individualized attribution scores and, therefore, remains necessary for clinical scenarios that focus on personalized risk explanation. Finally, our evaluation was conducted solely within the context of TJA complications using tree-based models. Although the theoretical rationale for PDPs aligns with population-guideline development principles that generalize beyond this specific domain, additional work is required to determine whether similar interpretability patterns apply to other diseases or modeling frameworks. Despite these limitations, this investigation provides a systematic demonstration of PDP’s validity over SHAP for clinical guideline development using a large TJA dataset analyzed for interaction effects, offering strong evidence that population-level interpretability methods are well-suited for translating machine learning insights into actionable clinical protocols.

## Conclusion

In summary, this analysis of 517,826 TJA cases demonstrates that while SHAP effectively ranks feature importance at the individual level, PDPs offer greater utility for population-level clinical decision-making by revealing dose–response relationships and actionable risk thresholds. Our threshold-based approach identified that interaction effects contributed 49.9% of the total model influence, with age × ASA classification and operative time × ASA classification being the most clinically significant synergistic risk combinations. These population-level patterns support the development of preoperative optimization strategies by identifying thresholds that remain consistent across the cohort. At the same time, several limitations should be considered when interpreting these findings. Both PDP and SHAP are post-hoc explanations of moderately performing models, and the PDP-derived thresholds presented here should be viewed as proof-of-concept rather than definitive clinical cutoffs. PDPs also estimate average marginal effects and cannot capture individualized prediction differences, limiting their application for patient-specific counseling. Although our findings are specific to TJA and the modeling framework used, they demonstrate how population-level interpretability tools like PDP can complement individualized SHAP explanations and collectively provide a systematic foundation for translating machine learning insights into guideline-oriented clinical protocols.

## Supplementary Information

Supplementary Material 1.

Supplementary Material 2.

## Data Availability

The datasets analyzed were obtained from the American College of Surgeons National Surgical Quality Improvement Program (ACS-NSQIP), which provides data access to qualified researchers through an application process. These data are not publicly available.
