# Supplementary material for: Evaluating the methodological suitability of partial dependence plots and Shapley additive explanations for population-level interpretation of machine learning models in total joint arthroplasty
Source: Arthroplasty. 2026 Jan 29;8:8. doi: 10.1186/s42836-025-00360-9 (PMC12853731; doi:10.1186/s42836-025-00360-9)
Supplement: Supplementary file 2 — Supplementary Material 1. [file 42836_2025_360_MOESM1_ESM.docx]

**Supplementary material**

**Table S1.** Baseline characteristics of training and test sets

| **Variable** | **Training Set (*n* = 414,260)** | **Test Set**  **(*n* = 103,566)** | **Absolute Difference** |
| --- | --- | --- | --- |
| **Continuous Variables (mean ± SD)** |  |  |  |
| Age, years | 67.0 ± 10.0 | 67.0 ± 10.0 | 0.02 |
| Body Mass Index, kg/m² | 31.8 ± 6.5 | 31.7 ± 6.5 | 0.04 |
| Preoperative Hematocrit, % | 41.4 ± 3.9 | 41.4 ± 3.9 | 0.003 |
| Preoperative Albumin, g/dL | 4.20 ± 0.29 | 4.20 ± 0.29 | 0.001 |
| Preoperative Creatinine, mg/dL | 0.91 ± 0.39 | 0.91 ± 0.40 | 0.001 |
| Hemoglobin A1c, % | 5.72 ± 0.29 | 5.72 ± 0.29 | 0.0003 |
| Operative Time, minutes | 90.9 ± 35.6 | 91.0 ± 35.6 | 0.05 |
| **Categorical Variables, *n* (%)** |  |  |  |
| Sex |  |  |  |
| Female | 241,560 (58.3%) | 60,389 (58.3%) | 0.002% |
| Male | 172,482 (41.6%) | 43,123 (41.6%) | 0.002% |
| Other/Unknown | 218 (0.05%) | 54 (0.05%) | 0.0005% |
| Diabetes Mellitus |  |  |  |
| No diabetes | 346,457 (83.6%) | 86,662 (83.7%) | 0.05% |
| Non-insulin dependent | 52,894 (12.8%) | 13,093 (12.6%) | 0.13% |
| Insulin-dependent | 14,909 (3.6%) | 3,811 (3.7%) | 0.08% |
| Current Smoking | 35,197 (8.5%) | 8,856 (8.6%) | 0.05% |
| Functional Status |  |  |  |
| Independent | 405,757 (97.9%) | 101,454 (98.0%) | 0.01% |
| Partially dependent | 5,552 (1.3%) | 1,329 (1.3%) | 0.06% |
| Totally dependent | 280 (0.07%) | 73 (0.07%) | 0.004% |
| Unknown | 2,671 (0.6%) | 710 (0.7%) | 0.04% |
| Hypertension | 248,394 (60.0%) | 62,050 (59.9%) | 0.05% |
| COPD | 14,163 (3.4%) | 3,572 (3.4%) | 0.03% |
| Congestive Heart Failure | 7,581 (1.8%) | 1,900 (1.8%) | 0.005% |
| Chronic Steroid Use | 16,579 (4.0%) | 4,177 (4.0%) | 0.03% |
| Weight Loss >10% |  |  |  |
| No | 162,433 (39.2%) | 40,807 (39.4%) | 0.19% |
| Yes | 252 (0.06%) | 62 (0.06%) | 0.001% |
| Unknown | 251,575 (60.7%) | 62,697 (60.5%) | 0.19% |
| Bleeding Disorder | 7,989 (1.9%) | 2,002 (1.9%) | 0.005% |
| ASA Physical Status Classification |  |  |  |
| ASA 0 | 7,677 (1.9%) | 1,891 (1.8%) | 0.03% |
| ASA 1 | 193,805 (46.8%) | 48,376 (46.7%) | 0.07% |
| ASA 2 | 203,831 (49.2%) | 51,095 (49.3%) | 0.13% |
| ASA 3 | 8,346 (2.0%) | 2,078 (2.0%) | 0.008% |
| ASA 4 | 25 (0.006%) | 9 (0.009%) | 0.003% |
| ASA 5 | 576 (0.14%) | 117 (0.11%) | 0.03% |
| Elective Surgery |  |  |  |
| Non-Elective | 3,429 (0.8%) | 860 (0.8%) | 0.0003% |
| Elective | 159,214 (38.4%) | 39,998 (38.6%) | 0.19% |
| Unknown | 251,617 (60.7%) | 62,708 (60.5%) | 0.19% |
| Total Hip Arthroplasty | 168,284 (40.6%) | 42,200 (40.7%) | 0.12% |
| Outcome Variable |  |  |  |
| 30-Day Complication | 27,612 (6.666%) | 6,953 (6.666%) | <0.001% |
