# Supplementary material for: Evaluating the methodological suitability of partial dependence plots and Shapley additive explanations for population-level interpretation of machine learning models in total joint arthroplasty
Source: Arthroplasty. 2026 Jan 29;8:8. doi: 10.1186/s42836-025-00360-9 (PMC12853731; doi:10.1186/s42836-025-00360-9)
Supplement: Supplementary file 3 — Supplementary Material 2. [file 42836_2025_360_MOESM2_ESM.docx]

**Supplemental material**

**Table S2.** List of 24 explicit interaction terms created for the interaction model analysis.

| **Variable 1** | **Variable 2** | **Interaction Term** |
| --- | --- | --- |
| Age (years) | ASA Physical Status Classification | Age × ASA |
| Operative Time (minutes) | ASA Physical Status Classification | Operative Time × ASA |
| Operative Time (minutes) | Age (years) | Operative Time × Age |
| Preoperative Hematocrit (%) | ASA Physical Status Classification | Hematocrit × ASA |
| Preoperative Hematocrit (%) | Diabetes Mellitus | Hematocrit × Diabetes |
| Body Mass Index (kg/m²) | Age (years) | BMI × Age |
| Diabetes Mellitus | Current Smoking | Diabetes × Smoking |
| Body Mass Index (kg/m²) | Diabetes Mellitus | BMI × Diabetes |
| Functional Status | Age (years) | Functional Status × Age |
| Current Smoking | Chronic Obstructive Pulmonary Disease | Smoking × COPD |
| Preoperative Hematocrit (%) | Age (years) | Hematocrit × Age |
| Body Mass Index (kg/m²) | ASA Physical Status Classification | BMI × ASA |
| Diabetes Mellitus | Age (years) | Diabetes × Age |
| Total Hip Arthroplasty | Age (years) | Procedure Type × Age |
| Hypertension | Age (years) | Hypertension × Age |
| Chronic Steroid Use | ASA Physical Status Classification | Steroid Use × ASA |
| Body Mass Index (kg/m²) | Current Smoking | BMI × Smoking |
| Operative Time (minutes) | Diabetes Mellitus | Operative Time × Diabetes |
| Diabetes Mellitus | Hypertension | Diabetes × Hypertension |
| Body Mass Index (kg/m²) | Hypertension | BMI × Hypertension |
| Age (years) | Current Smoking | Age × Smoking |
| Chronic Steroid Use | Diabetes Mellitus | Steroid Use × Diabetes |
| Congestive Heart Failure | Age (years) | CHF × Age |
| Weight Loss >10% | Age (years) | Weight Loss × Age |
